# Abnormalities of the placenta

**DOI:** 10.1186/1471-2393-12-S1-A2

**Published:** 2012-08-28

**Authors:** Alexander Heazell

**Affiliations:** 1 University of Manchester, UK

## 

Normal placental structure and function is essential for a healthy pregnancy; the placenta is responsible for nutrient and oxygen transport, removal of waste products, protection from infection, modulation of the maternal immune system and hormone production to maintain pregnancy. The human placenta is structurally adapted to fulfil this role as it is haemomonochorial, minimising the distance between maternal and fetal circulations to maximise exchange. Disorders of the placenta including: FGR, pre-eclampsia, placental abruption and abnormal (velamentous) cord insertion are associated with over 50% of stillbirths and are frequently cited as the primary cause of death [1-3].

Abnormal placental structure and function significantly increases the risk of stillbirth. Levels of pregnancy associated plasma protein A (PAPP-A) in the lowest 5% and alpha fetoprotein (AFP) in the highest 5% increase the risk of stillbirth by 50-fold and 2.8-fold respectively [4,5]. In women at high-risk of pregnancy complications, abnormal placental structure and/or blood flow seen by ultrasound scan at 19-23 weeks preceded 15 out of 22 stillbirths [6]. There are few studies of microscopic placental structure in stillbirth. However, microscopic structure and cell turnover of the villous trophoblast are disrupted in FGR and pre-eclampsia [7-9]. Evidence from genetic analyses demonstrated that gene imprinting in the placenta is altered in pregnancy loss [10]. The importance of placenta genetics and epigenetics is supported by the observation of increased stillbirth and pregnancy loss in confined placental mosaicism where genetic abnormalities are only present in the placenta [11].

Due to its central role in determining pregnancy outcome, detailed examination of the placenta can give useful information about the cause of stillbirth and is recommended by the Royal College of Obstetricians and Gynaecologists (RCOG), American College of Obstetricians and Gynecologists (ACOG) and Perinatal Society of Australia and New Zealand (PSANZ) [12,13]. Examination of the placenta reduces the proportion of unexplained stillbirths [14]. Consequently, examination of the placenta is one of the most common investigations undertaken after a stillbirth and is one of the most valuable [2,15].

Despite the value of placental examination to identify conditions associated with stillbirth including: fetal thrombotic vasculopathy, chronic intervillositis, villitis, chorioamnionitis, funisitis, infarction, massive perivillous fibrin deposition, villous dysmaturity (increased syncytial knot formation), villous immaturity and cord lesions further research is needed to increase the understanding of placental pathology in stillbirth. This is reflected in the research priorities for high-income countries synthesised by an international panel of experts which mention need for repositories of well-phenotyped samples from stillbirths and from well-matched controls and the need to understand the pathophysiological pathways in common conditions associated with stillbirth including: diabetes, cigarette smoking and maternal obesity [16].

An important goal of translational medicine is to use the increased understanding of placental dysfunction to develop improved tests of pregnancy wellbeing. For example, factors derived from the placenta, such as human placental lactogen or placental growth factor may provide novel means to identify pregnancies at highest risk of stillbirth [17]. Discovery-based technologies such as proteomics or metabolomics offer the opportunity to analyse biofluids and tissue in hypothesis-generating studies to provide a more holistic understanding of fetal and placental dysfunction. Better understanding of the role of the placenta in stillbirth offers the opportunity to develop strategies to identify placental dysfunction in pregnancies, in order that intervention may be targeted to prevent stillbirth.
